# Thermal and Cardiovascular Strain Mitigate the Potential Benefit of Carbohydrate Mouth Rinse During Self-Paced Exercise in the Heat

**DOI:** 10.3389/fphys.2015.00354

**Published:** 2015-11-25

**Authors:** Matthew N. Cramer, Martin W. Thompson, Julien D. Périard

**Affiliations:** ^1^School of Human Kinetics, Faculty of Health Sciences, University of OttawaOttawa, ON, Canada; ^2^Discipline of Exercise and Sport Science, Faculty of Health Sciences, University of SydneyLidcombe, NSW, Australia; ^3^Athlete Health and Performance Research Centre, Aspetar Orthopaedic and Sports Medicine HospitalDoha, Qatar

**Keywords:** hyperthermia, perceived exertion, exercise performance, cycling, time trial, fatigue

## Abstract

**Purpose:** To determine whether a carbohydrate mouth rinse can alter self-paced exercise performance independently of a high degree of thermal and cardiovascular strain.

**Methods:** Eight endurance-trained males performed two 40-km cycling time trials in 35°C, 60% RH while swilling a 20-ml bolus of 6.5% maltodextrin (CHO) or a color- and taste-matched placebo (PLA) every 5 km. Heart rate, power output, rectal temperature (T_re_), and mean skin temperature (T_sk_) were recorded continuously; cardiac output, oxygen uptake (VO_2_), mean arterial pressure (MAP), and perceived exertion (RPE) were measured every 10 min.

**Results:** Performance time and mean power output were similar between treatments, averaging 63.9 ± 3.2 and 64.3 ± 2.8 min, and 251 ± 23 and 242 ± 18 W in CHO and PLA, respectively. Power output, stroke volume, cardiac output, MAP, and VO_2_ decreased during both trials, increasing slightly or remaining stable during a final 2-km end-spurt. T_re_, T_sk_, heart rate, and RPE increased throughout exercise similarly with both treatments. Changes in RPE correlated with those in T_re_ (*P* < 0.005) and heart rate (*P* < 0.001).

**Conclusions:** These findings suggest that carbohydrate mouth rinsing does not improve ~1-h time trial performance in hot-humid conditions, possibly due to a failure in down-regulating RPE, which may be influenced more by severe thermal and cardiovascular strain.

## Introduction

During prolonged exercise (≥90 min) in temperate conditions, carbohydrate ingestion increases time to fatigue by maintaining a high rate of carbohydrate oxidation and sparing muscle glycogen (Coyle et al., 1986; Tsintzas et al., 1996). Performance of shorter-duration exercise (~1 h) is also improved by carbohydrate ingestion in both temperate (el-Sayed et al., 1997; Jeukendrup et al., 1997; Millard-Stafford et al., 1997; Desbrow et al., 2004) and hot (Below et al., 1995) conditions; however, the ergogenic effect may be of non-metabolic origin, as performance improvements have been observed despite similar carbohydrate availability (Carter et al., 2004b), glycogen oxidation rates (Hawley et al., 1997; McConell et al., 2000), and muscle glycogen storage (Bosch et al., 1996; Hawley et al., 1997).

In the absence of any direct metabolic effect, carbohydrate ingestion may directly influence the central nervous system during exercise. Central fatigue, defined as a progressive reduction in voluntary muscle activation (Gandevia, 2001), may be delayed by carbohydrate ingestion via stimulation of oral carbohydrate receptors, activating brain regions associated with motivation and pleasure, and affecting behavior and possibly exercise performance (Carter et al., 2004a). In temperate conditions, use of a carbohydrate mouth rinse, which isolates a potential central effect, results in improved endurance performance by 1.9–11.6% (Jeukendrup, 2013), characterized by higher running speed (Rollo et al., 2008, 2010) and cycling power output (Carter et al., 2004a; Chambers et al., 2009; Pottier et al., 2010; Fares and Kayser, 2011; Lane et al., 2013), despite similar heart rates and ratings of perceived exertion (RPE). This latter finding suggests that a carbohydrate mouth rinse may down-regulate RPE, permitting a higher self-selected work rate for a given sense of effort. Nevertheless, some studies report no effect of carbohydrate mouth rinsing on performance (Whitham and McKinney, 2007; Beelen et al., 2009).

Carbohydrate mouth rinsing has also shown to provide no additional benefit to performance during time trial exercise under heat stress (Che Muhamed et al., 2014; Watson et al., 2014). However, the reason why mouth rising is ineffective in improving self-paced exercise performance in the heat remains unclear. Since any intervention that influences RPE can alter performance, the perceptual benefits associated with a carbohydrate mouth rinse may be negated in adverse environmental conditions. Indeed, the attainment of high core and skin temperatures, and the attendant cardiovascular strain, is a major limitation to time trial performance in the heat (Ely et al., 2010; Périard et al., 2011; Périard and Racinais, 2015a,b). In such conditions, greater skin blood flow requirements demand a redistribution of cardiac output (Rowell, 1974) without compromising oxygen delivery to active muscles (González-Alonso et al., 2008). Nevertheless, maximum oxygen uptake (VO_2max_) decreases during high-intensity exercise in hot/humid conditions (Arngrímsson et al., 2003; González-Alonso and Calbet, 2003; Arngrímsson et al., 2004a; Wingo et al., 2005), resulting in a higher relative exercise intensity for a given external workload. Consequently, power output is reduced during time trial efforts in the heat, while RPE remains similar or is exacerbated, relative to exercise performed in cooler conditions (Tatterson et al., 2000; Tucker et al., 2004; Périard et al., 2011; Périard and Racinais, 2015a,b). As relative exercise intensity potentiates sensations of effort (Sargeant and Davies, 1973; Skinner et al., 1973; Robertson, 1982), self-selected workload is likely to be reduced for a given RPE in the heat. Accordingly, a thermoregulatory-mediated increase in cardiovascular strain is likely to counteract the potential influence of a carbohydrate mouth rinse regimen on down-regulating RPE and ultimately improving time trial performance in the heat. To isolate the role of cardiovascular strain on potentially mitigating the influence of a carbohydrate mouth rinse in down-regulating RPE during exercise-heat stress, the factors characterizing the cardiovascular response must be elucidated in parallel with changes in performance.

Therefore, the present study investigated whether a carbohydrate mouth rinse can alter 40-km time trial performance (~1 h) among trained cyclists in hot ambient conditions, independently of a high degree of thermal and cardiovascular strain. It was hypothesized that a carbohydrate mouth rinse would not improve self-paced performance of ~1 h, as afferent inputs related to thermal and cardiovascular strain would overwhelm any potential ergogenic benefit of a carbohydrate mouth rinse in down-regulating the sense of effort.

## Methods

### Subjects

Eight well-trained cyclists completed the experimental protocol. Age, mass, height, maximum heart rate, and VO_2max_ were 31.4 ± 4.1 y, 75.3 ± 6.3 kg, 1.76 ± 0.06 m, 182 ± 11 beats min^−1^, and 65.8 ± 6.5 ml·kg^−1^·min^−1^, respectively. All participants were cycling a minimum of 250 km·wk^−1^ for 6 week prior to preliminary testing and were well-practized in time trial competitions. Experiments were performed in the Southern Hemisphere winter when participants were not naturally acclimatized to the heat. Before experimentation, participants were fully informed of the potential risks and provided written consent. This study was approved by the Human Research Ethics Committee of the University of Sydney and conformed to guidelines set forth by the Declaration of Helsinki.

### General procedures

The experimental protocol required three visits to the laboratory. Visit 1 was a preliminary session in which anthropometric measurements were taken and an incremental cycling protocol to exhaustion was completed to determine VO_2max_. Visits 2–3 were experimental trials during which participants completed simulated 40-km cycling time trials in an environmental chamber maintained at 35°C and 60% relative humidity (RH) while facing an air velocity of 2.8 m·s^−1^. Each participant was given either a maltodextrin (CHO) or placebo (PLA) solution to rinse their mouth at fixed distance intervals. Prior to these experimental visits, participants were told that each solution was thought to augment cycling performance. Treatments were administered in a double-blinded, counterbalanced order, and each time trial was separated by 5–7 days. All exercise testing was performed on an SRM cycle ergometer (Schoberer Rad Meßtechnik, Jülich, Germany) at the same time of day to avoid any potential effect of circadian variation. Participants wore only cycling shorts, shoes, and socks during all trials.

During the 24-h period preceding preliminary testing, participants were encouraged to abstain from caffeine, alcohol, and high-intensity exercise. A final meal was consumed 3 h prior to testing and, participants were given 300 ml of water upon arrival at the laboratory. Participants were permitted to consume water *ad libitum* during all testing sessions to replicate their normal hydration strategies. A record of diet and activity was kept by each participant, and was replicated 24 h prior to all subsequent testing sessions.

### Preliminary session

During the preliminary session, height and body mass were measured using a precision stadiometer and scale (Mettler ID1, Greitensee, Switzerland). Participants then entered the environmental chamber, which was set to 20°C and 40% RH. Handlebar and saddle positions were adjusted according to each participant's preference and comfort and recorded for future trials. By fitting the ergometer settings to each participant, coupled with the performance of a preliminary exercise test, each participant was able to become accustomed with the equipment prior to experimentation. Participants then performed four 5-min submaximal stages (100, 150, 200, and 250 W) followed by an incremental protocol to determine VO_2max_, consisting of increasing external workloads (25 W·min^−1^) until volitional exhaustion. Samples of expired gases were collected over 1 min using the Douglas bag method and analyzed using zirconium cell oxygen (PM1111E Servomex Sugar Land, TX) and carbon dioxide (IrI507 Servomex, Sugar Land, TX) analysers. Both analysers were calibrated against gases of known composition (βOC, Chatswood, Australia). Expired gas volumes were measured using a dry gas meter (Harvard, Kent, UK) and corrected to STPD.

### Experimental trials

For experimental trials, participants arrived ~60 min prior to testing. Upon arrival, each participant voided his bladder, changed into cycling attire, and inserted a rectal thermistor probe. After body mass was then measured, participants sat for 20 min for instrumentation with skin temperature sensors and a heart rate transmitter (Polar T31, Kempele, Finland). Participants entered the climate chamber, were seated on the cycling ergometer, and the first bolus of a mouth rinse was administered. They were then instructed to complete a distance of 40 km as quickly as possible. No feedback was provided with regard to time, wattage, cadence or heart rate. Upon reaching the penultimate kilometer, subjects were asked to finish the time trial at maximal effort while measures of heart rate, VO_2_ and cardiac output were taken. Encouragement was given during the final kilometer to ensure maximal effort was maintained.

### CHO solution

Participants swilled a 20-ml bolus of 6.5 g·100 ml^−1^ maltodextrin solution or a placebo at 5-km intervals of the time trial, which is in line with previous studies in both hot (Che Muhamed et al., 2014; Watson et al., 2014) and cool (Chambers et al., 2009; Fares and Kayser, 2011) conditions. The placebo solution was matched for color (green) and taste (lime) using a sugar-free cordial/water mixture. During the time trial, the solutions were kept outside the environmental chamber at an ambient temperature of 22°C. The participants were instructed to swill the solution in their mouth for 5 s and to then expectorate it into a separate container. They were reminded to avoid swallowing any solution. Following the completion of the second time trial participants were made aware of the true composition of each solution.

### Cardiovascular and temperature measurement

Expired gases were collected at 10-min intervals to calculate VO_2_ as in the preliminary testing session (i.e., Douglas bag method). Cardiac output was measured via carbon dioxide re-breathing (Collier, 1956) at 10-min intervals, and stroke volume was calculated using the Fick equation. Following the measurement of VO_2_, rebreathing was performed using an anesthesia bag filled with a gas mixture containing 12% CO_2_. Rebreathing lasted ~15 s, which is the time taken for CO_2_ equilibrium to be attained across the bag, alveoli and pulmonary arterial blood. During this time an exponential capnograph tracing was produced. As soon as the capnograph tracing captured CO_2_ equilibrium, the rebreathing valve was closed and the mouth-piece removed. A sphygmomanometer was used to measure mean arterial pressure (MAP) at 10-min intervals (Accoson, Essex, England). Continuous measurements of T_re_ were made using a calibrated thermistor probe (YSI 400, Mallinkrodt Medical, Yellow Springs, OH) inserted 12 cm beyond the anal sphincter that were recorded on a portable data logger (T-Logger, University of Sydney, Australia). Skin temperature was measured and recorded continuously using iButton® temperature sensors (Maxim Integrated Products, Sunnyvale, CA). Both the T-Logger and iButton® sensors were calibrated prior to and following completion of the study using a water bath of temperatures ranging from 15 to 50°C with calculated accuracies of ± 0.05°C and ± 0.01°C, respectively. For T_sk_, a four-point area-weighted mean was calculated (Ramanathan, 1964). RPE was recorded at 10-min intervals.

### Hematological measurements

Venous blood samples were drawn immediately prior to exercise and upon completion of exercise. Blood collection was performed using 2 ml heparinised capillary tubes. Plasma glucose and lactate concentrations were analyzed in duplicate using the glucose oxidase and lactate oxidase method, respectively (EML 105, Radiometer Pacific, Copenhagen, Denmark). Reported values represent the mean of the duplicate measurements.

### Statistical analysis

All statistical analyses were performed using Prism 6 (GraphPad Software, Inc., San Diego, CA). A two-way repeated measures analysis of variance was performed using the independent factors of treatment and time. Effect size was measured using partial eta-squared (η^2^) values with η^2^ > 0.06 representing a moderate effect and η^2^ ≥ 0.14 a large effect. Variables independent of time were analyzed with a paired Student's *t*-test. A linear mixed model analysis was used to evaluate the relationship between RPE as the dependent variable and T_re_ and heart rate as indicators of thermal and cardiovascular strain, respectively. Alpha was set at the *p* < 0.05 level. All data are expressed as means ± standard deviations (SD).

## Results

### Performance

**Figure 1:**
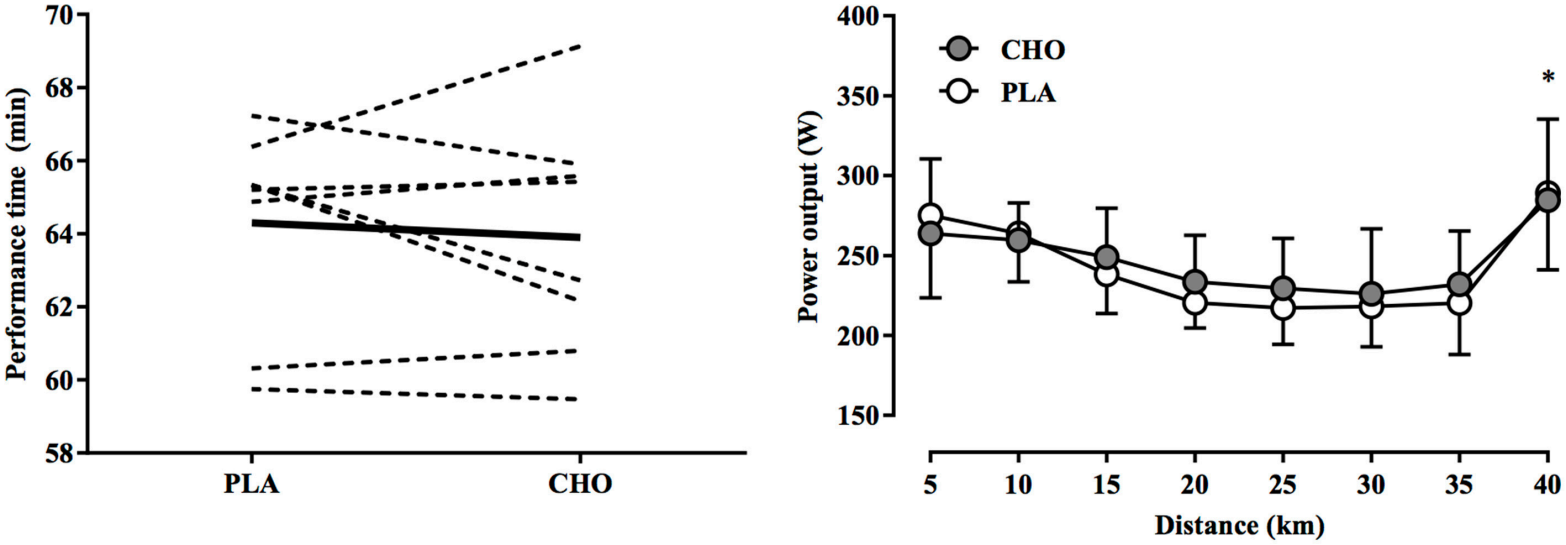
**Individual differences in performance time (left) and mean power output at 5-km intervals (right) during a 40-km cycling time trial in hot and humid conditions while swilling either a 6.5% carbohydrate solution (CHO) relative to performance time with a placebo solution (PLA) matched for color and taste**. For individual performance times, thin dashed and thick black lines indicate individual and mean differences in performance time, expressed as a absolute change from the PLA treatment, respectively. No significant treatment-by-time interaction was observed; ^*^ indicates main effect of time (*P* < 0.01).

Mean completion time was 63.9 ± 3.2 min and 64.3 ± 2.8 min in the CHO and PLA trials, respectively (*P* = 0.57). Compared to PLA, time trial performance in CHO was 0.62 ± 2.93% faster, with four participants demonstrating better performance with CHO and four with PLA (Figure 1). Completion time was also similar between the first (64.3 ± 3.3 min) and second (63.9 ± 2.6 min) time trials (*P* = 0.62). With both treatments, power output declined progressively, reached a nadir at 30 km, and then increased rapidly during the 2-km end-spurt with both treatments (Figure 1). While a main effect of time was observed for power output (*P* < 0.01, η^2^ = 0.351), no significant treatment-by-time interaction was evident (*P* = 0.77, η^2^ = 0.001).

### Cardiovascular and temperature responses

**Figure 2:**
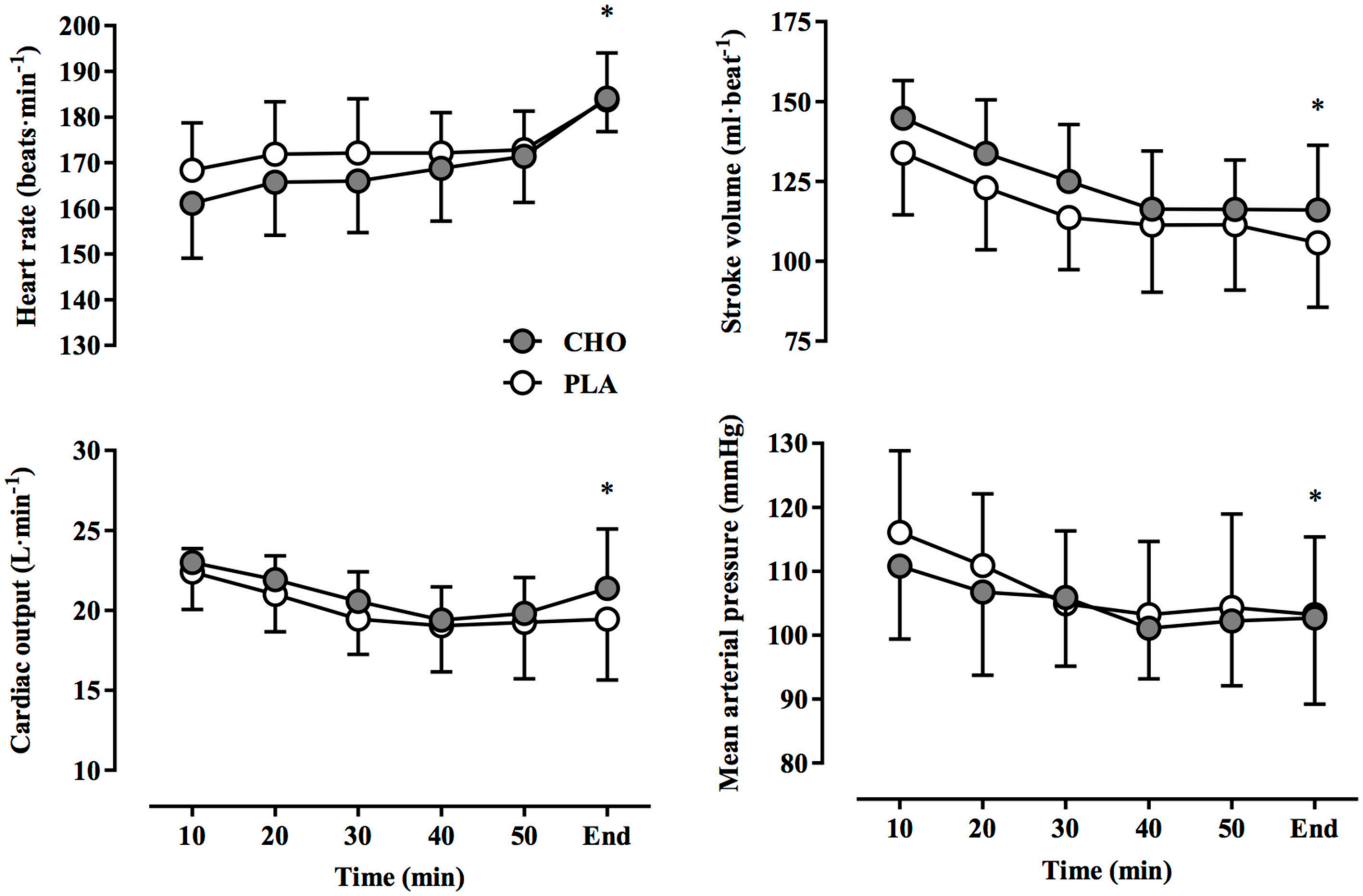
**Heart rate, stroke volume, cardiac output, and mean arterial pressure at 10-min intervals and at the end of exercise during a 40-km cycling time trial in hot and humid conditions while rinsing the oral cavity with a carbohydrate solution (CHO) or taste- and color-matched placebo (PLA)**. No significant treatment-by-time interaction was observed; ^*^ indicates main effect of time (*P* < 0.01).

**Figure 3:**
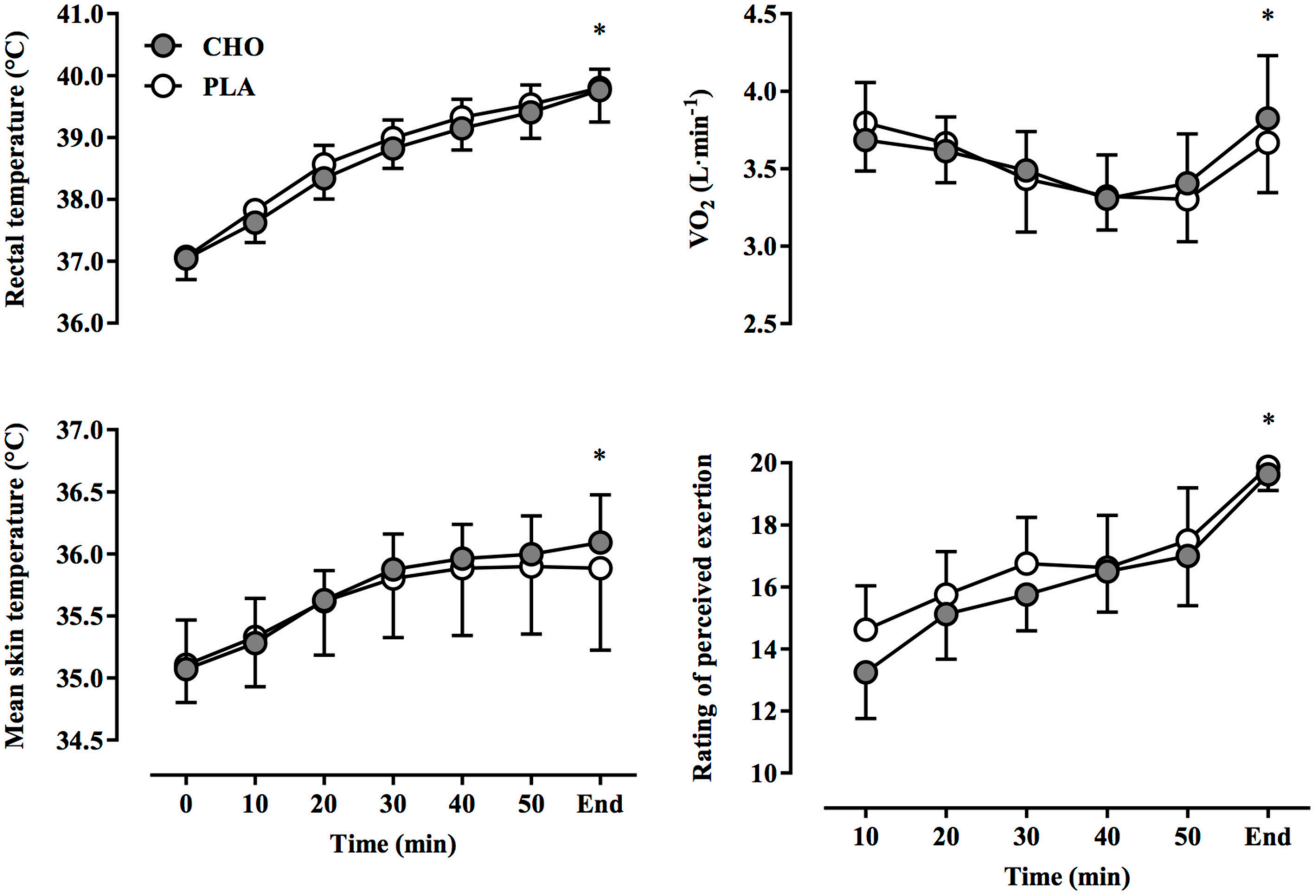
**Rectal and mean skin temperatures, rate of oxygen uptake (VO_2_), and ratings of perceived exertion (RPE) at 10-min intervals and at the end of exercise during a 40-km cycling time trial in hot and humid conditions while rinsing the oral cavity with a carbohydrate solution (CHO) or taste- and color-matched placebo (PLA)**. No significant treatment-by-time interaction was observed; ^*^ indicates main effect of time (*P* < 0.01).

Cardiovascular responses are presented in Figure 2. Heart rate increased over time, reaching final values of 184 ± 3 beats·min^−1^ and 184 ± 4 beats·min^−1^ in CHO and PLA trials, respectively (*P* < 0.01, η^2^ = 0.258). Cardiac output, stroke volume, and MAP fell progressively over time (*P* < 0.01, η^2^ = 0.113). No treatment-by-time interactions were found for heart rate (*P* = 0.18, η^2^ = 0.014), stroke volume (*P* = 0.76, η^2^ = 0.005), cardiac output (*P* = 0.85, η^2^ = 0.009), and MAP (*P* = 0.87, η^2^ = 0.008). Values for VO_2_ declined gradually from 10 to 50 min (Figure 3), but increased rapidly toward the end of exercise during the end-spurt; however, no treatment-by-time interaction was found (*P* = 0.65, η^2^ = 0.019). Both T_re_ and T_sk_ increased over time (*P* < 0.01, η^2^ = 0.406), but no difference between treatments was evident for either T_re_ (*P* = 0.85, η^2^ = 0.001) or T_sk_ (*P* = 0.87, η^2^ = 0.006) (Figure 3). Since initial performance time was similar with CHO and PLA, the rate of change in T_re_ was also not different between conditions (CHO: 0.04 ± 0.01°C·min^−1^, PLA: 0.04 ± 0.01°C·min^−1^; *P* = 0.92).

### Perceptual, hydration, and hematological responses

**Figure 4:**
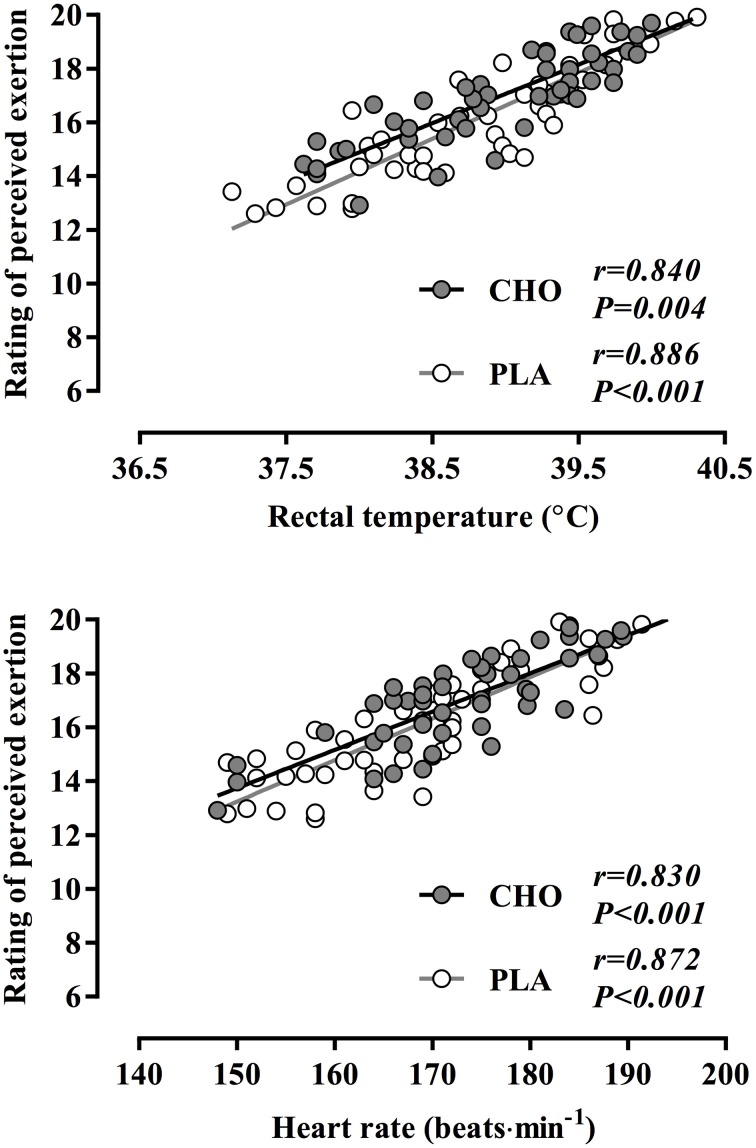
**Rating of perceived exertion plotted against rectal temperature (i.e., thermal strain) and heart rate (i.e., cardiovascular strain) during a 40-km cycling time trial in hot and humid conditions while rinsing the oral cavity with a carbohydrate solution (CHO) or taste- and color-matched placebo (PLA)**.

Over time, RPE increased significantly with both CHO and PLA treatments (*P* < 0.01, η^2^ = 0.64), yet no significant treatment-by-time interaction was evident (*P* = 0.38, η^2^ = 0.009) (Figure 3). At the completion of the time trial, RPE values reached an average of 19.6 ± 0.2 and 19.9 ± 0.1 in CHO and PLA trials, respectively (*P* = 0.18). RPE was strongly correlated to changes in T_re_ (*P* < 0.005) and heart rate (*P* < 0.001) in both CHO and PLA treatments (Figure 4). Participants consumed 1.27 ± 0.18 L and 1.49 ± 0.24 L of water during CHO and PLA trials, respectively, which was similar between treatments (*P* = 0.49). The mean change in body mass was not different between trials (CHO: −0.82 ± 0.33%, PLA: −0.74 ± 0.39%; *P* = 0.88). Blood glucose was similar at rest (CHO: 5.02 ± 0.27 mmol·L^−1^, PLA: 5.04 ± 0.27 mmol·L^−1^; *P* = 0.92) and at the end of exercise (CHO: 5.48 ± 0.29 mmol·L^−1^, PLA: 5.55 ± 0.40 mmol·L^−1^; *P* = 0.87). Blood lactate concentration rose during exercise, but resting (CHO: 1.76 ± 0.22 mmol·L^−1^, PLA: 1.57 ± 0.12 mmol·L^−1^; *P* = 0.47) and end-exercise (CHO: 5.50 ± 0.79 mmol·L^−1^, PLA: 6.02 ± 0.73 mmol·L^−1^; *P* = 0.57) values were similar between treatments.

## Discussion

The main finding of this study is that afferents originating from high thermal (i.e., T_re_ and T_sk_) and cardiovascular (i.e., heart rate and MAP) strain appear to counteract any potential ergogenic effect of a CHO mouth rinse in the heat. Indeed, rinsing the oral cavity with a 20-ml bolus of 6.5% maltodextrin solution (CHO) for 5 s at 5-km intervals did not improve 40-km cycling time trial performance among well-trained cyclists compared to a taste- and color-matched placebo (PLA) in hot-humid conditions. This is in agreement with previous observations demonstrating no influence of a CHO mouth rinse on performance in warm/hot conditions (Che Muhamed et al., 2014; Watson et al., 2014). However, our data extend these observations to suggest that since the exercise duration, distance covered, constitution of the mouth rinse, and duration of rinsing were similar to those of previous studies showing performance improvements in temperate conditions (Carter et al., 2004a; Chambers et al., 2009; Fares and Kayser, 2011), the lack of any ergogenic effect in the CHO vs. PLA treatment indicates factors unrelated to oral carbohydrate sensation may be more potent modulators of perceived exertion and performance in the heat. This observation is reinforced by the relationship between RPE and T_re_, as well as RPE and heart rate in both conditions (Figure 4).

In most studies carbohydrate mouth rinsing improved self-paced exercise performance of 30–60 min duration in temperate environmental conditions (17–22°C, 45–62% RH), despite similar levels of physiological (i.e., heart rate) and perceptual (i.e., RPE) strain compared to a placebo treatment (Carter et al., 2004a; Rollo et al., 2008, 2010; Chambers et al., 2009; Pottier et al., 2010; Lane et al., 2013). In the present study, self-paced exercise in hot-humid conditions (35°C, 60% RH) resulted in similar physiological and perceptual responses (Figures 2, 3) between CHO and PLA treatments, yet no differences in performance time and power output were found (Figure 1). Recent investigations using similarly trained participants in warm environmental conditions have produced comparable results. For example, carbohydrate mouth rinsing during 30 min of submaximal exercise resulted in no performance benefit over a taste-matched placebo during a subsequent 10-km time trial in 32°C and 75% RH (Che Muhamed et al., 2014). While the lack of any benefit of CHO in that study may be explained by the relatively short time trial duration (~12 min), Watson et al. (2014) recently found that total work completed during a 1-h time trial in 30°C, 60% RH conditions was not enhanced by carbohydrate mouth rinsing vs. placebo. However, the diminished effectiveness of the mouth rinse on performance in the heat was not elucidated. Therefore, the performance data presented herein confirm previous findings and extends these to suggest that a thermal strain-mediated increase in physiological strain may be more potent in modulating perceived exertion and performance in the heat. This is underscored by the similar rise in T_re_ and T_sk_ (Figure 3), along with the concomitant development of cardiovascular strain (Figure 2) between conditions, which demonstrates the dominant influence of these factors on performance (Figure 1) and RPE (Figure 4).

The ergogenic effect of carbohydrate mouth rinsing in temperate climatic conditions appears to operate via stimulation of oral carbohydrate receptors that activate brain regions associated with reward, resulting in greater self-selected exercise intensity for a given RPE (Chambers et al., 2009). While this may have been the case, the lack of any subsequent performance improvement in our hot-humid conditions suggests that afferent sensory input from the muscles, heart, respiratory system and thermoreceptors (Ray and Gracey, 1997; Kayser, 2003; Schepers and Ringkamp, 2009) may have counteracted this response. When core and skin temperatures are sufficiently elevated, competition for cardiac output may result between cutaneous and active muscle vasculature to facilitate internal heat transfer to the skin while maintaining arterial blood pressure (Rowell et al., 1966; Rowell, 1974; González-Alonso et al., 2008). The displacement of blood to the periphery reduces cardiac pre-load, cardiac output, MAP, and VO_2max_ (Arngrímsson et al., 2003; González-Alonso and Calbet, 2003; Arngrímsson et al., 2004a; Wingo et al., 2005; Périard et al., 2011), the latter of which has been shown to decline by ~19% during steady-state cycling (Wingo et al., 2005) and ~17% during self-paced cycling in the heat (Périard et al., 2011; Périard and Racinais, 2015a,b). In the present study, T_re_ and T_sk_ rose throughout exercise while power output declined contemporaneously with stroke volume, cardiac output, and MAP, which reached a nadir in the final stages of the time trial. Additionally, VO_2_ in the final minute of the time trial reached values similar those achieved during the first 10 min of exercise, representing a ~24% reduction compared to values attained in preliminary testing. Moreover, changes in these variables over time were similar between treatments, and no differences in RPE were observed. Recent investigations demonstrating the ineffectiveness of carbohydrate mouth rinsing in the heat measured neither haemodynamic responses nor VO_2_; however, end-exercise core temperature values were ≥39.0°C, mean skin temperatures were >34.0°C, heart rate exceeded 180 beats·min^−1^, and RPE was similar between carbohydrate and placebo mouth rinses (Che Muhamed et al., 2014; Watson et al., 2014). The notion that elevated thermal strain (i.e., a narrow core-to-skin temperature gradient), which increases skin blood flow requirements and concomitantly the cardiovascular response, is the primary factor impairing submaximal aerobic exercise performance in the heat (Sawka et al., 2012), is supported by studies in which pre-cooling decreased thermal and cardiovascular strain, along with the perception of thermal discomfort early during exercise to permit faster overall time trial performances (Arngrïmsson et al., 2004b; Faulkner et al., 2015). Therefore, we speculate that a high degree of thermal and cardiovascular strain, particularly toward the end of the time trial, may have completely mitigated any possible ergogenic effect of a carbohydrate mouth rinse in our study and others.

The relationship between self-selected exercise intensity and RPE has been a topic of considerable focus. Perceived exertion may modulate performance by means of self-determined fluctuations in external workload in order to prevent an “unsustainable” RPE that would be deleterious to performance (Tucker, 2009). In this model, the integration of a variety of internal (e.g., muscle glycogen) and external (e.g., ambient temperature) signals would provide the necessary sensory information to formulate an RPE. The oral presence of carbohydrate may fit into this model as a signal of metabolic status as performance improvements of ~2% have been demonstrated following carbohydrate ingestion (el-Sayed et al., 1997; Jeukendrup et al., 1997), and of up to 11.6% with carbohydrate mouth rinsing in the fasted state (Fares and Kayser, 2011), despite similarities in RPE. Therefore, RPE in these studies may have been effectively “down-regulated” for a given absolute workload. Despite similar performance time, power output, and RPE between treatments, profound levels of thermal and cardiovascular strain were evident in the present study, suggesting that high T_re_, T_sk_, and/or a reduced MAP are more potent contributors to RPE and performance than the oral presence of carbohydrate. This is supported by the strong correlation noted between RPE and the increase in T_re_, as well as the rise in heart rate (Figure 4). The interaction between factors contributing to RPE may be altered under different environmental conditions (Pandolf, 1982); specifically, RPE is more strongly associated with relative compared to absolute VO_2_ values (Sargeant and Davies, 1973) and correlates positively with core temperature (Nybo and Nielsen, 2001b). Therefore, it is possible that under hot-humid conditions the perception of effort may have been influenced to a greater extent by thermal and cardiovascular strain, resulting in impaired oxygen delivery (González-Alonso and Calbet, 2003) and a higher relative exercise intensity (Arngrímsson et al., 2004a), thereby overwhelming the possible ergogenic effect of a carbohydrate mouth rinse. Under these conditions, the primary afferent signal(s) to RPE may be hyperthermia and/or the attending cardiovascular strain. Accordingly, RPE is often exacerbated during self-paced exercise in the heat (Tatterson et al., 2000; Périard et al., 2011; Périard and Racinais, 2015a,b), despite the maintenance of a similar or even slightly lower relative exercise intensity than in cooler conditions (Périard and Racinais, 2015b). Interestingly, this occurs in conjunction with a reduced cerebral blood flow (Périard and Racinais, 2015a), which has been suggested to exacerbate central fatigue due to inadequate oxygen delivery to the brain (Nybo and Nielsen, 2001a; Nybo and Rasmussen, 2007; Rasmussen et al., 2007). The development of fatigue during intensive exercise however, with or without hyperthermia, is associated with an enhanced cerebral metabolism (González-Alonso et al., 2004) characterized by a compensatory increase in oxygen extraction in the brain upon reaching volitional exhaustion (Trangmar et al., 2014). Thus, it is unlikely that reductions in cerebral blood flow and oxygenation *per se* influence RPE through the development of central fatigue. Notwithstanding, a limitation of the current study is the lack of experimental time trials conducted in temperate conditions. Future studies should consider conducting trials in various environmental conditions to better isolate the potential influence of CHO mouth rinsing on RPE and performance.

In summary, rinsing the oral cavity with a carbohydrate solution did not influence RPE nor improve 40-km time trial performance in hot-humid conditions. It would appear that additional afferents originating from high thermal and cardiovascular strain may have counteracted any possible ergogenic effect of a CHO mouth rinse in the heat. Hence, strategies that attenuate the rise in core and skin temperature may be more efficacious in optimizing performance, as high thermal and cardiovascular strain likely offset the potential benefits of a carbohydrate mouth rinse.

## Author contributions

MC, MT, and JP conceived and designed the experiments. JP and MC collected, analyzed, and interpreted the data. MC drafted and revised the manuscript. JP and MT revised the manuscript. MC, MT, and JP approved the final version of the manuscript.

### Conflict of interest statement

The authors declare that the research was conducted in the absence of any commercial or financial relationships that could be construed as a potential conflict of interest.
